# Safety and immunogenicity of three doses of non-typeable *Haemophilus influenzae*-*Moraxella catarrhalis* (NTHi-Mcat) vaccine when administered according to two different schedules: a phase 2, randomised, observer-blind study

**DOI:** 10.1186/s12931-022-02019-4

**Published:** 2022-05-04

**Authors:** Ilaria Galgani, Margherita Annaratone, Daniela Casula, Gennaro Di Maro, Michel Janssens, Annaelisa Tasciotti, Tino Schwarz, Murdo Ferguson, Ashwani Kumar Arora

**Affiliations:** 1 GSK, Siena, Italy; 2 GSK, Rixensart, Belgium; 3 Institute of Laboratory Medicine and Vaccination Centre, Klinikum Würzburg Mitte, Campus Juliusspital, Würzburg, Germany; 4 Colchester Research Group, Truro, Canada

**Keywords:** Non-typeable *Haemophilus influenzae*, *Moraxella catarrhalis*, COPD, Exacerbation, Vaccination, Immunogenicity, Safety

## Abstract

**Background:**

Non-typeable *Haemophilus influenzae* (NTHi) and *Moraxella catarrhalis* (Mcat) infections are frequently associated with exacerbations of chronic obstructive pulmonary disease (COPD). Results were reported with a two-dose (0–2 months) schedule of an investigational AS01_E_-adjuvanted NTHi-Mcat vaccine containing three surface proteins from NTHi and one from Mcat. We evaluated the safety and immunogenicity of three NTHi-Mcat vaccine doses administered in two different schedules to adults with a smoking history (≥ 10 pack-years), immunologically representing the COPD population.

**Methods:**

In this 18-month, randomised (1:1), observer-blind study with 6-month open follow-up, 200 healthy adults aged 40–80 years received NTHi-Mcat vaccine at 0–2–6 months and placebo at 12 months (0–2–6 group), or vaccine at 0–2–12 months and placebo at 6 months (0–2–12 group). Solicited and unsolicited adverse events (AEs) were recorded for 7 and 30 days, respectively, post-vaccination, and potential immune-mediated diseases (pIMDs) and serious AEs (SAEs) throughout the study. Immune responses were assessed.

**Results:**

No safety concerns were identified with the third vaccine dose or overall. Most solicited AEs were mild/moderate. Unsolicited AEs were reported in 16%, 16.1% and 14.4% of participants in the 0–2–6 group post-dose 1, 2 and 3, respectively, and 20%, 20.4% and 9.7%, respectively, in the 0–2–12 group. In 24 months, SAEs were reported in 12 participants in the 0–2–6 group and 9 in the 0–2–12 group (18 events in each group). There were three deaths (unknown cause, 0–2–6 group; myocardial infarction, lung cancer in 0–2–12 group). pIMDs were reported in three participants in the 0–2–6 group (non-serious inflammatory bowel disease, gout, psoriasis) and three in the 0–2–12 group (serious ulcerative colitis, two with non-serious gout). The SAEs, deaths and pIMDs were considered not causally related to vaccination. Antigen-specific antibody concentrations were higher at 12 months post-dose 1 with the 0–2–6 schedule than with the 0–2–12 schedule and at 12 months post-dose 3 were similar between schedules, remaining higher than baseline.

**Conclusions:**

No safety concerns were identified when the investigational NTHi-Mcat vaccine was administered via a 0–2–6 months or 0–2–12 months schedule to older adults with a smoking history. Persistent immune responses were observed after the third vaccine dose.

*Trial registration* https://clinicaltrials.gov/; NCT03443427, registered February 23, 2018.

**Supplementary Information:**

The online version contains supplementary material available at 10.1186/s12931-022-02019-4.

## Background

Chronic obstructive pulmonary disease (COPD) is a syndrome with increasing prevalence worldwide [1] that results from numerous interacting factors, with smoking the strongest inciting feature [2, 3]. Acute exacerbations of COPD (AECOPD) are a major cause of hospital admission and readmission and can have severe negative impacts for patients with COPD, including higher mortality rates, shorter long-term survival and poorer quality of life when compared to those without readmissions [4].

There is increasing evidence that the lung microbiome plays a key role in AECOPD [5]. Non-typeable *Haemophilus influenzae* (NTHi) and *Moraxella catarrhalis* (Mcat) are bacterial pathogens that are frequently associated with AECOPD [6–9] and their presence in sputum samples in stable COPD is associated with a higher risk of a subsequent Hi- or Mcat-associated exacerbation compared with earlier absence [10]. Moreover, there is evidence that chronic NTHi infection fuels airway inflammation [11] and that NTHi and Mcat act as co-pathogens in COPD [12–14].

An investigational adjuvanted multi-component vaccine was developed to reduce the frequency of AECOPD, containing four conserved surface proteins involved in the virulence mechanisms of NTHi and Mcat: three from NTHi, a free recombinant protein D (PD) and a recombinant fusion protein combining protein E and Pilin A (PE-PilA), and the fourth from Mcat, ubiquitous surface protein A2 (UspA2) [15]. A phase 1 study of the NTHi-Mcat vaccine showed no safety concerns and antigen-specific immune responses in adults given two doses 60 days apart [15]. Similar findings were previously reported in studies of a vaccine containing only the NTHi components [16]. Adults aged 18–40 years were recruited, followed by older adults who were current or former smokers to immunologically represent the COPD population [15, 16], given evidence that smoking can alter the immune system before COPD is recognised [17–19].

In the assessment of the NTHi vaccine, formulations that included the Adjuvant System AS01_E_ [20] produced the highest humoral and cellular immune responses in older adults [16]. AS01_E_-adjuvanted NTHi-Mcat vaccine formulations were therefore administered to older adults with a smoking history in the phase 1 study [15]. The formulation containing 10 µg PD, 10 µg PE-PilA and 3.3 µg UspA2 administered as two doses, 60 days apart, induced the best humoral response against the tested antigens and was evaluated in a subsequent randomised, observer-blind, placebo-controlled phase 2b trial, in patients with moderate to very severe COPD [21]. Again, there were no safety concerns with the NTHi-Mcat vaccine and vaccination was immunogenic. However, there was no evidence of efficacy in reducing the yearly rate of moderate/severe exacerbations, although there was some suggestion of a reduced frequency of severe exacerbations and related hospitalisations following vaccination [21].

The present study evaluated the safety of administering three doses of the NTHi-Mcat vaccine to older adults with a history of smoking, with the first two doses given 60 days apart and the third given 6 or 12 months after the first. The persistence of immune responses against each NTHi and Mcat antigen over 24 months was also assessed.

**Fig. 1:**
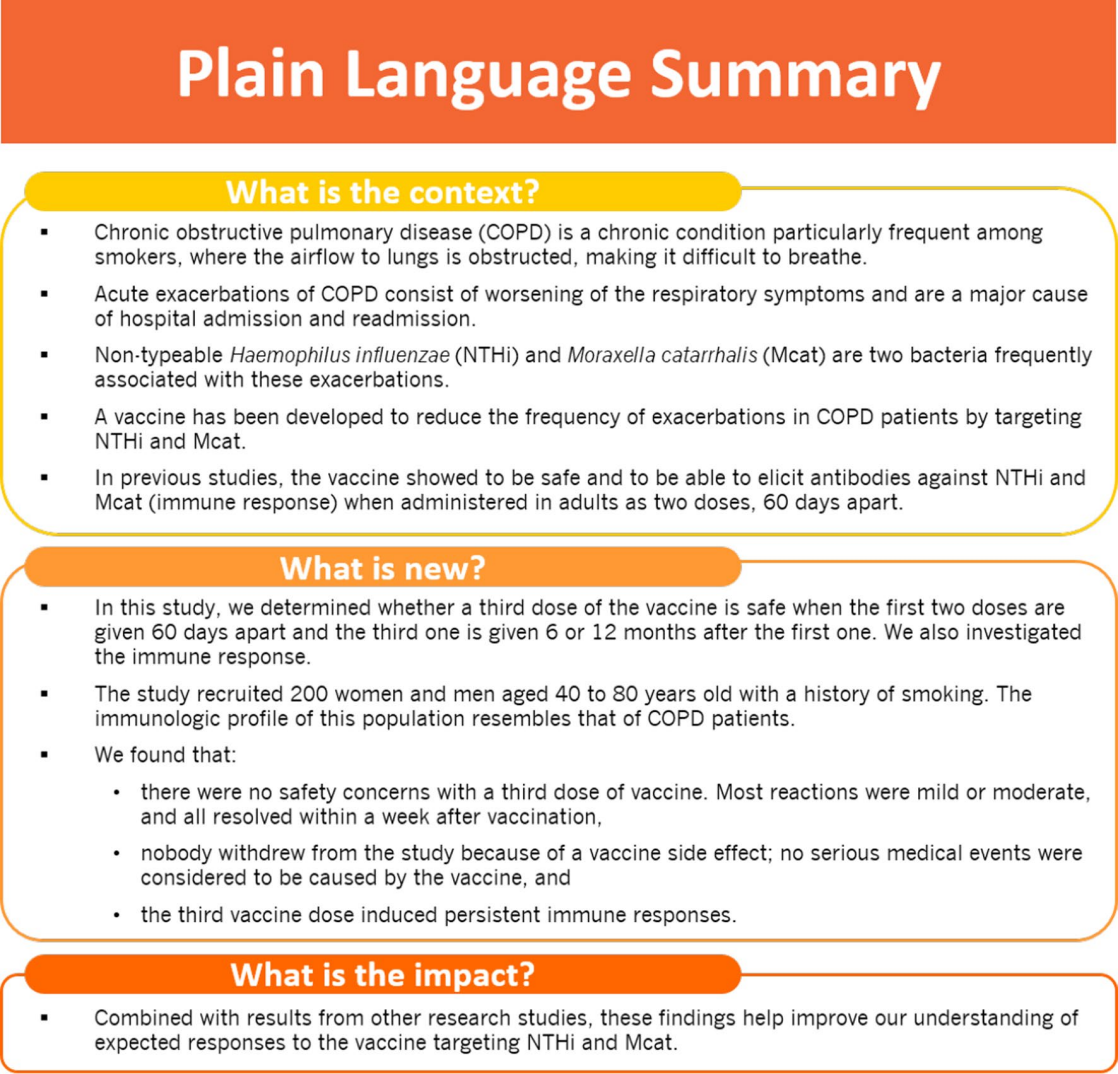
Plain language summary

Figure 1 summarises the clinical relevance of this study for the COPD population.

## Methods

### Study design and participants

**Fig. 2:**
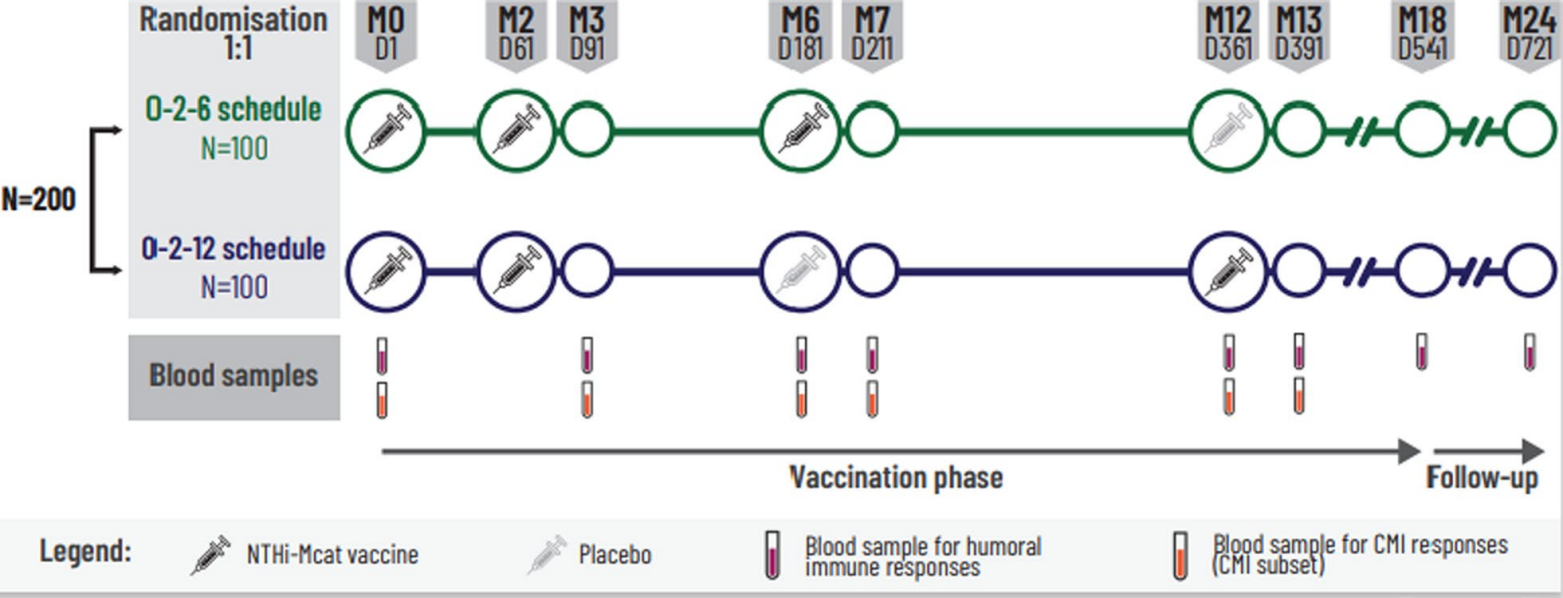
Study design. CMI, cell-mediated immunity; D, study day; M, study month; N, number of participants; Mcat, *Moraxella catarrhalis*; NTHi, non-typeable *Haemophilus influenzae*

This phase 2, randomised, observer-blind study was conducted in eight centres in three countries (Canada, Germany and the UK) between March 2018 and September 2020 (ClinicalTrials.gov identifier: NCT03443427). The observer-blind study, which lasted 18 months (vaccination phase), was followed by a 6-month open follow-up period (Fig. 2). A study summary is available at www.gsk-studyregister.com (study identifier, 207759).

Adults aged 40–80 years with a smoking history of at least 10 pack-years were administered four intramuscular injections: three injections of the AS01_E_-adjuvanted NTHi-Mcat vaccine containing 10 µg of each NTHi antigen (PD and PE-PilA) and 3.3 µg of the Mcat antigen (UspA2) and one injection of placebo (saline solution). The vaccine antigens were described previously [15, 16]. AS01_E_ is an adjuvant system containing 3-*O*-desacyl-4'-monophosphoryl lipid A (MPL), *Quillaja saponaria* Molina, fraction 21 (QS-21; licensed by GSK from Antigenics LLC, a wholly owned subsidiary of Agenus Inc., a Delaware USA corporation) and liposome (25 μg MPL and 25 µg QS-21) [20]. All study inclusion and exclusion criteria are provided in the online supplementary methods.

Participants were randomly assigned (1:1 ratio) to one of two study groups. One group received three doses of NTHi-Mcat vaccine at Days 1, 61 and 181, and a placebo injection at Day 361 (0–2–6 months schedule; 0–2–6 group) and the second group received two doses of NTHi-Mcat vaccine at Days 1 and 61, a placebo injection at Day 181, and a third NTHi-Mcat vaccine dose at Day 361 (0–2–12 months schedule; 0–2–12 group) (Fig. 2). Randomisation of supplies was performed at GSK using MATerial Excellence (MATEX), a program developed by GSK for use with Statistical Analysis Systems software (SAS; Cary, NC, USA). Participants were allocated to a study group at each site via a central randomisation system. Due to differences in the appearance of the study vaccine and the placebo formulation for the third and fourth injections, and because the vaccine was prepared and reconstituted on site, the study was conducted in an observer-blind manner, i.e. formulation recipients and those responsible for the evaluation of any study endpoint were unaware of whether a third vaccine dose or placebo was administered, and each formulation was prepared and administered by authorised medical personnel who did not participate in any of the study’s clinical evaluations.

The primary objective of the study was to assess the safety and reactogenicity of the investigational NTHi-Mcat vaccine administered in a 0–2–6 or 0–2–12 months schedule and secondary objectives were to evaluate the vaccine’s long-term safety and its humoral and cellular immunogenicity. The study was conducted in accordance with the Declaration of Helsinki and Good Clinical Practice. The protocols and associated documents were reviewed and approved by the ethics committee of each participating centre. All participants provided written informed consent before study entry.

### Safety and reactogenicity

Solicited local (pain, redness and swelling at the injection site) and general (fatigue, gastrointestinal symptoms, headache, myalgia, chills and fever) adverse events (AEs) were recorded for 7 days after each injection and unsolicited AEs for 30 days after each injection on diary cards. In addition, all participants were contacted by telephone 7 days after each injection and any safety concerns during the previous 7 days were recorded in the electronic case report form. AE intensity was graded on a 0–3 scale. Grade 3 (severe) intensity was defined as redness or swelling of diameter > 100 mm, temperature ≥ 39.0 °C and, for all other AEs, prevention of normal activities. Data on potential immune-mediated diseases (pIMDs) [22], serious AEs (SAEs) and AEs leading to withdrawal were collected throughout the 24-month study.

### Humoral and cellular immunogenicity

Blood samples were collected for the assessment of humoral immunogenicity before the first, third and fourth injections (Days 1, 181 and 361), 30 days after the second, third and fourth injections (Days 91, 211 and 391) and 18 and 24 months after the first injection (Days 541 and 721) (Fig. 2). Immunoglobulin G antibody levels to each vaccine antigen were measured by enzyme-linked immunosorbent assay (ELISA), developed and qualified by GSK. Sera were stored at − 20 °C or below until assayed. Standardised procedures and in-house-made reference serum were used for each assay. The assay cut-off (lower limit of quantification) was 153 ELISA units (EU)/mL, 16 EU/mL, 8 EU/mL and 28 EU/mL for anti-PD, anti-PE, anti-PilA and anti-UspA2, respectively.

This humoral immune response evaluation was complemented with an investigation of cell-mediated immunity (CMI), specifically, antigen-specific T cells. The CMI subset was selected from sites able to process blood samples according to GSK procedures for peripheral blood mononuclear cell preparation. Blood samples for CMI analysis were taken before the first, third and fourth injections and 30 days after the second, third and fourth injections (Fig. 2) in a subset of participants (around 20 per group). CMI responses (antigen-specific CD4^+^ and CD8^+^ T cells) were measured by flow cytometry using intracellular cytokine staining on peripheral blood mononuclear cells, following an adaptation of previously described methods [23]. The gating strategy for measuring antigen-specific T cells is described in the online supplementary methods (Additional file 1: Fig. S1). No CD8^+^ T cell response was observed. Numbers of antigen-specific CD4^+^ T cells expressing, upon stimulation, at least two different markers among CD40 ligand (CD40L), interleukin (IL)-2, tumour necrosis factor (TNF)-α, interferon (IFN)-ɣ, IL-13 and IL-17 were calculated.

### Statistical analysis

A sample size of 100 participants in each group allowed a two-sided 95% confidence interval (CI) with width equal to 0.165, calculated using the Clopper–Pearson CI formula [24], assuming that 20% of participants would experience at least one grade 3 symptom following a third vaccine dose. With this sample size, the probability of observing at least one serious or severe event with incidence of 1.5% was 95%.

The safety analysis was performed on the total vaccinated cohort, including all vaccinated participants. The incidence of AEs per study group was calculated with exact 95% CIs on all safety data after each injection up to 18 months after the first vaccine dose and on all safety data derived from the 6-month follow-up period ending 24 months after the first vaccine dose.

The immunogenicity analysis was performed on the per-protocol immunogenicity cohort, including eligible adults who received the study vaccine as specified in the protocol and complied with study procedures and for whom post-vaccination immunogenicity results were available for at least one antigen. Antibody geometric mean concentrations (GMCs) were determined with 95% CIs. Post-dose 1 GMCs were calculated using an ANCOVA model including pre-dose 1 antibody concentration as covariate and treatment, country, age category and smoking status as factors, while pre-dose 1 GMCs were calculated via an ANOVA including treatment, country, age category and smoking status as factors. Geometric mean ratios (GMRs) with 95% CIs were calculated to describe the change in antibody concentration at a specific time point with respect to the antibody concentration at an earlier time point (‘baselines’, i.e. pre-dose 1, pre-dose 3 and pre-dose 4). GMRs were calculated using an ANOVA on the log_10_ ratio between antibody concentrations at specific time points and previous log_10_ antibody concentrations at an earlier time point, with treatment, country, age category and smoking status as factors. Descriptive statistics were used to summarise the frequency, by study group, of specific CD4^+^ T cells expressing at least two cytokine markers.

The primary endpoint was related to safety and planned to be descriptive only (i.e. no treatment comparison with regards to safety was planned). No alpha adjustment for multiple testing of the secondary endpoints (immunogenicity) was performed; CIs and differences between schedules should be interpreted with caution.

The statistical analyses were performed using SAS within the Life Science Analytics Framework system version 9.4.

## Results

### Study population

**Fig. 3:**
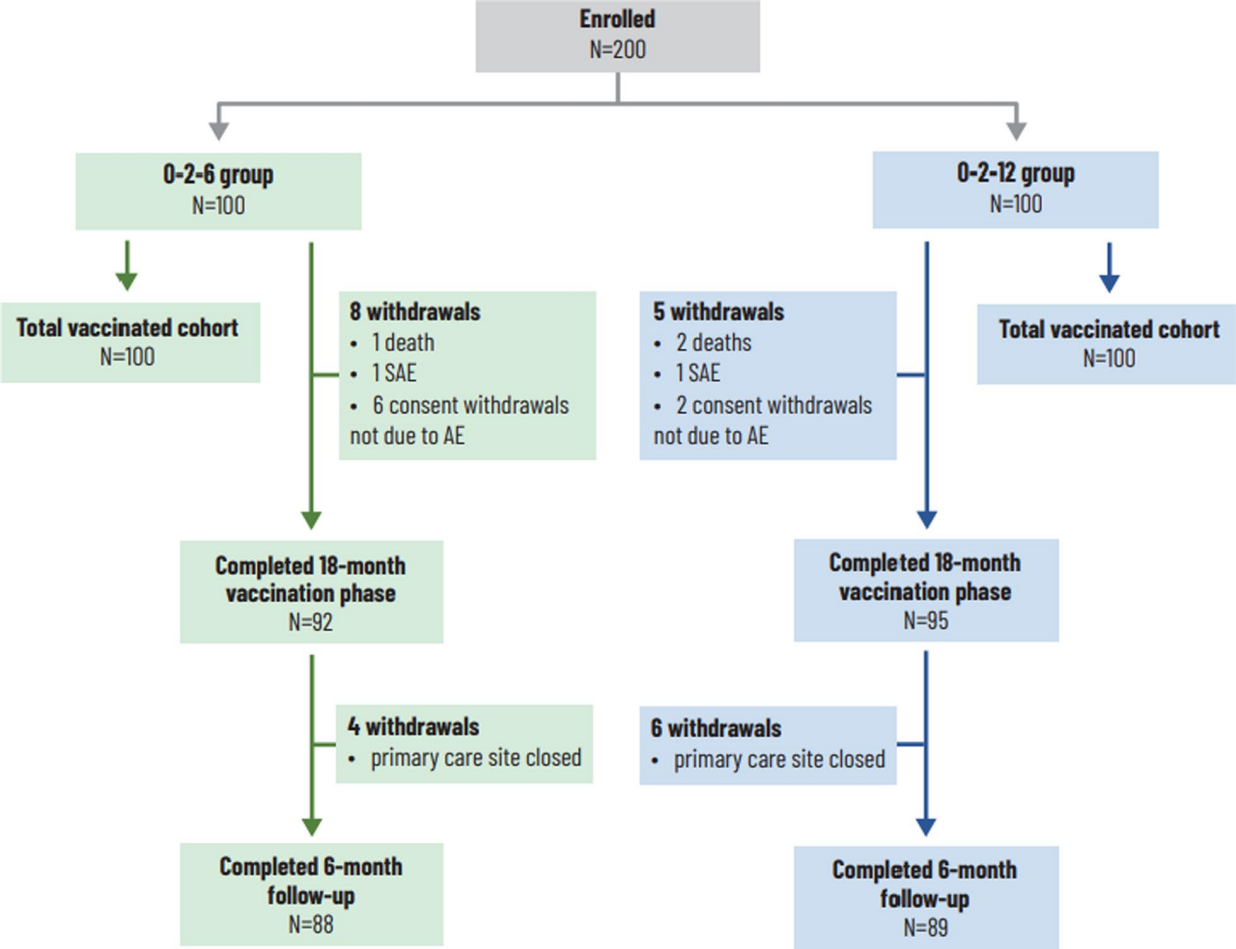
Disposition of the study participants and reasons for withdrawal. N, number of participants; AE, adverse event; SAE, serious adverse event (considered not causally related to study vaccination). 0–2–6 group was given vaccine at 0–2–6 months and placebo at 12 months; 0–2–12 group was given vaccine at 0–2–12 months and placebo at 6 months

Two hundred adults with a smoking history were enrolled and received at least one dose of the NTHi-Mcat vaccine; 90 participants in the 0–2–6 group and 93 in the 0–2–12 group received three doses. A total of 187 participants completed the 18-month vaccination phase; reasons for withdrawal are shown in Fig. 3. During the 6-month follow-up, 10 participants withdrew because a primary care site closed for research. Baseline characteristics of the participants were comparable between the groups (Table 1).

**Table 1:** Characteristics of the study participants at enrolment (total vaccinated cohort)

Characteristic	0–2–6 schedule (N = 100)	0–2–12 schedule (N = 100)
Age (years) at dose 1, mean (SD)	58.4 (10.3)	59.8 (10.1)
Age range, n (%)		
18–64 years	68 (68.0)	65 (65.0)
65–84 years	32 (32.0)	35 (35.0)
Female sex, n (%)	46 (46.0)	43 (43.0)
Race, n (%)		
White	100 (100)	99 (99.0)
Native American/Alaska native	0	1 (1.0)
Smoking status, n (%)		
Current smoker	66 (66.0)	54 (54.0)
Former smoker	34 (34.0)	46 (46.0)
Pack-years, mean (SD)	36.7 (17.2)	32.5 (16.8)

N, number of participants; n, number of participants in a specific category; SD, standard deviation. 0–2–6 schedule, group given vaccine at 0–2–6 months and placebo at 12 months; 0–2–12 schedule, group given vaccine at 0–2–12 months and placebo at 6 months

### Safety and reactogenicity

**Fig. 4:**
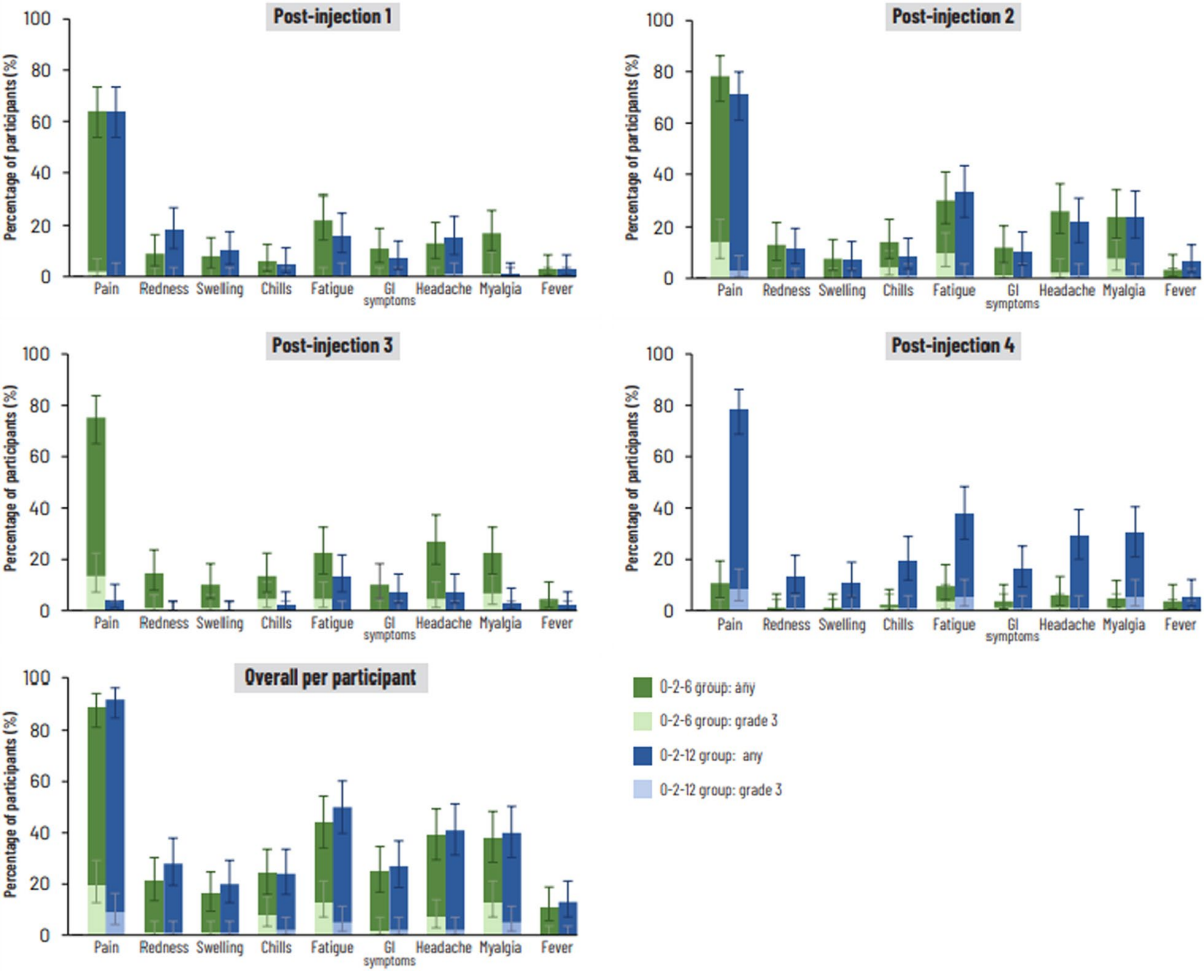
Percentages of participants (with exact 95% confidence intervals) reporting solicited local (pain, redness and swelling) and general (fatigue, gastrointestinal symptoms, headache, myalgia, chills and fever) adverse events (any intensity and grade 3 intensity) during the 7-day period after each injection and overall per participant (total vaccinated cohort). Injections 1 and 2, vaccine doses in both groups; injection 3, vaccine dose in 0–2–6 group, placebo in 0–2–12 group; injection 4, placebo in 0–2–6 group, vaccine dose in 0–2–12 group. GI (gastrointestinal) symptoms defined as nausea, vomiting, diarrhoea and/or abdominal pain. Fever defined as temperature ≥ 37.5 °C. Grade 3 intensity defined as redness or swelling of diameter > 100 mm, temperature ≥ 39 °C and, for all other adverse events, prevention of normal activities. Number of participants: 100, 92, 89 and 84 post-injection 1, 2, 3 and 4, respectively, for 0–2–6 group; 100, 97, 97, 93, respectively, for 0–2–12 group

The percentage of participants in the 0–2–6 group reporting a solicited local AE during the 7-day post-injection period was 67%, 78.3% and 77.5% after the first, second and third vaccine doses, respectively, and 10.7% after the placebo injection at Month 12. In the 0–2–12 group, the percentage reporting a solicited local AE was 69%, 73.2% and 79.6% after the first, second and third vaccine doses, respectively, and 4.1% after the placebo injection at Month 6. Pain was the most frequent solicited local AE overall, reported by 89% of participants in the 0–2–6 group and 92% of participants in the 0–2–12 group, and the most frequent solicited local AE after each vaccine dose (Fig. 4). Most solicited local AEs were mild or moderate (Fig. 4). Grade 3 pain was reported by 20% of participants in the 0–2–6 group (2%, 14.1% and 13.5% after vaccine dose 1, 2 and 3, respectively; no reports after placebo) and 9% of the 0–2–12 group (1%, 3.1% and 8.6% after vaccine dose 1, 2 and 3, respectively; no reports after placebo). All grade 3 solicited local AEs resolved within 5 days.

In the 0–2–6 group, 37%, 41.3% and 39.3% of participants reported a solicited general AE within 7 days after vaccine dose 1, 2 and 3, respectively, and 14.3% reported a solicited general AE after the placebo injection. In the 0–2–12 group, the percentage of participants reporting solicited general AEs was 35%, 47.4% and 53.8% after vaccine dose 1, 2 and 3, respectively, and 20.6% after placebo. The most frequent solicited general AE was fatigue overall (reported by 44% of participants in 0–2–6 group and 50% in 0–2–12 group) and after each NTHi-Mcat dose, apart from post-dose 1 in the 0–2–12 group (fatigue and myalgia each reported by 16% of participants) and post-dose 3 in the 0–2–6 group (headache, reported by 27% of participants) (Fig. 4). Most solicited general AEs were mild or moderate (Fig. 4). Overall, the most frequently reported grade 3 solicited general AEs were fatigue (reported by 13% and 5% of participants in 0–2–6 group and 0–2–12 group, respectively) and myalgia (13% and 5%, respectively). After the third NTHi-Mcat vaccine dose, the percentage of participants reporting grade 3 fatigue was 4.5% in the 0–2–6 group and 5.4% in the 0–2–12 group. Grade 3 myalgia was reported by 6.7% of participants in the 0–2–6 group and 5.4% in the 0–2–12 group after the third vaccine dose. There were no reports of grade 3 fever (temperature ≥ 39 °C). All grade 3 solicited general AEs resolved within 6 days.

At least one unsolicited AE was reported within 30 days of any injection in 38% of participants in the 0–2–6 group and 40% of participants in the 0–2–12 group (Table 2). The most frequently reported unsolicited AE was nasopharyngitis in both groups (Table 2). After each vaccine dose, unsolicited AEs were reported in 16%, 16.1% and 14.4% of participants in the 0–2–6 group post-dose 1, 2 and 3, respectively, and 20%, 20.4% and 9.7%, respectively, in the 0–2–12 group. After the placebo injection, unsolicited AEs were reported in 8.2% of the 0–2–6 group and 11.3% of the 0–2–12 group. Unsolicited AEs considered to be causally related to vaccination were reported in 7% of participants in both groups (11 reports in 0–2–6 group and 7 in 0–2–12 group); no unsolicited AE category (Medical Dictionary for Regulatory Activities preferred term) was reported more than once. Grade 3 unsolicited AEs considered causally related to vaccination were reported in one participant in the 0–2–6 group (ear discomfort and limb discomfort post-dose 3) and three participants in the 0–2–12 group (injection site pruritus and peripheral swelling post-dose 2, injection site pruritis post-dose 3).

**Table 2:** Percentage of participants reporting unsolicited adverse events (AEs) during the 30-day period after each injection given via the 0–2–6 months schedule (vaccine doses at 0–2–6 months and placebo at 12 months) and the 0–2–12 months schedule (vaccine doses at 0–2–12 months and placebo at 6 months) (total vaccinated cohort)

	**Percentage of participants (95% CI)**	
	**0–2–6 schedule (N = 100)**	**0–2–12 schedule (N = 100)**
At least one unsolicited AE (overall)	38.0 (28.5–48.3)	40.0 (30.3–50.3)
Related to vaccination	7.0 (2.9–13.9)	7.0 (2.9–13.9)
Grade 3 intensity	5.0 (1.6–11.3)	9.0 (4.2–16.4)
Grade 3 and related to vaccination	1.0 (0.0–5.4)	3.0 (0.6–8.5)
At least one unsolicited AE (by injection)		
First injection at 0 months	16.0 (9.4–24.7)	20.0 (12.7–29.2)
Second injection at 2 months	16.1 (9.3–25.2)	20.4 (12.9–29.7)
Third injection at 6 months	14.4 (7.9–23.4)	11.3 (5.8–19.4)
Fourth injection at 12 months	8.2 (3.4–16.2)	9.7 (4.5–17.6)
Unsolicited AEs reported in ≥ 4% of participants in at least one group		
Nasopharyngitis	6.0 (2.2–12.6)	6.0 (2.2–12.6)
Back pain	2.0 (0.2–7.0)	4.0 (1.1–9.9)
Hypertension	0	4.0 (1.1–9.9)

N, number of participants; 95% CI, 95% confidence interval

During the entire study, including the 6-month follow-up period, at least one SAE was reported in 12 participants (12%) in the 0–2–6 group and 9 (9%) in the 0–2–12 group (18 events in each group), none of which was assessed as causally related to study vaccination. Two of the SAEs (hemiparesis and pneumonia aspiration) were reported in two participants in the 6-month follow-up period (0–2–12 group). There was one death with unknown cause (participant collapsed and could not be resuscitated; cause of death unknown) that occurred in the 0–2–6 group 506 days after the first vaccine dose. There were two deaths in the 0–2–12 group, one due to myocardial infarction (239 days after first dose) and the other due to metastatic lung cancer (symptoms from 64 days after first dose). Two other events led to withdrawal from the study: lung cancer in one participant in the 0–2–6 group and breast cancer in one participant in the 0–2–12 group.

Seven pIMD occurrences were reported in six participants. In the 0–2–6 group, two non-serious psoriasis episodes were reported in a participant 5 and 15 days post-dose 1, and there was one report of non-serious gout 20 days post-dose 1 and one report of non-serious inflammatory bowel disease 192 days post-dose 3. In the 0–2–12 group, non-serious gout was reported in two participants 31 and 32 days after the placebo injection and one participant reported serious ulcerative colitis 159 days post-dose 1. By the end of the study, all pIMD events were resolving or had resolved, apart from the case of psoriasis. None of the deaths, withdrawals or pIMD cases were considered related to study vaccination.

### Immunogenicity

**Fig. 5:**
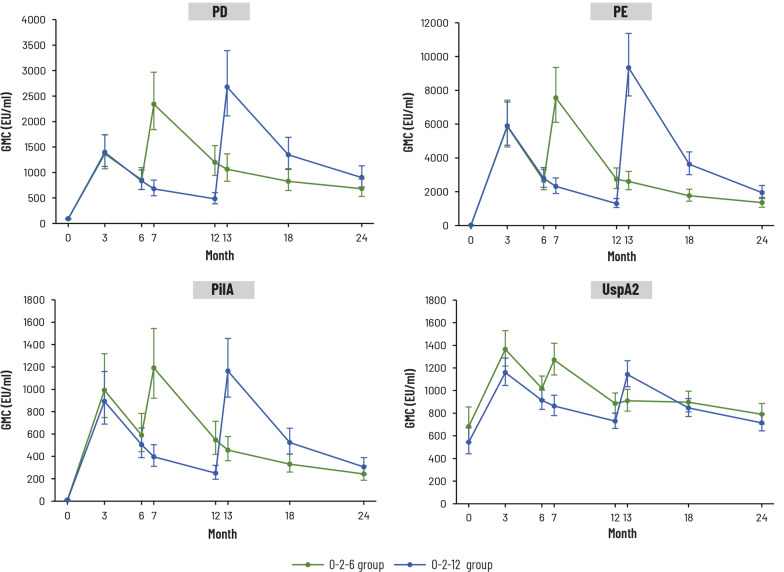
Adjusted geometric mean concentrations (GMCs with 95% confidence intervals) of antibodies against non-typeable *Haemophilus influenzae* antigens (PD, PE, PilA) and *Moraxella catarrhalis* antigen (UspA2) (per-protocol immunogenicity cohort). EU, ELISA units; PD, protein D; PE, protein E; PilA, Pilin A; UspA2, ubiquitous surface protein A2. 0–2–6 group was given vaccine at 0–2–6 months and placebo at 12 months; 0–2–12 group was given vaccine at 0–2–12 months and placebo at 6 months. Number of participants with available results at each time point: between 77 and 82 in 0–2–6 group, between 79 and 87 in 0–2–12 group

At baseline, antibody GMCs for each NTHi antigen were negligible, while the antibody GMC for Mcat UspA2 was 682.4 EU/mL in the 0–2–6 group and 544.9 EU/mL in the 0–2–12 group (Fig. 5). Antibody GMCs against each vaccine antigen peaked 1 month after two vaccine doses, declined up until the third vaccine dose time point and peaked again 1 month after the third vaccine dose in both groups (Fig. 5). Antibody GMCs at 12 months post-dose 1 were higher in the 0–2–6 group than in the 0–2–12 group for all antigens. Antibody GMCs 6 months post-dose 3 (Month 12 with 0–2–6 schedule and Month 18 with 0–2–12 schedule) and 12 months post-dose 3 (Month 18 and Month 24, respectively) were similar between groups (Fig. 5). At Month 24 (18 months post-dose 3 for 0–2–6 schedule and 12 months post-dose 3 for 0–2–12 schedule), antibody GMCs remained above baseline for all antigens in both groups (Fig. 5) and 93.5% to 100% of participants in both groups had seropositive antibody concentrations for each antigen (Additional file 1: Table S1).

**Fig. 6:**
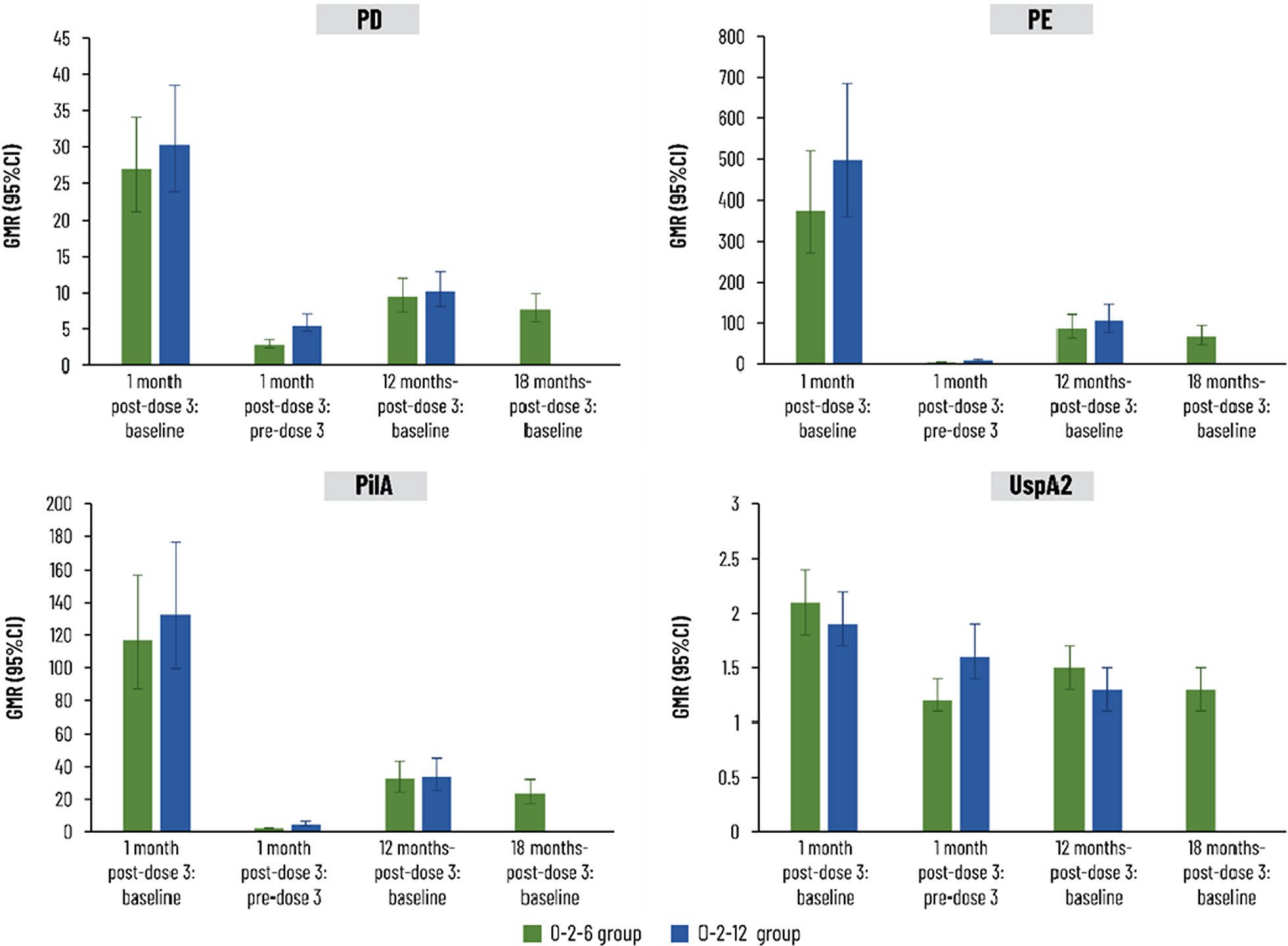
Geometric mean ratio (GMR) of log_10_ ratio of antibody concentrations at time points after the third vaccine dose versus baseline or immediately before the third dose was administered (pre-dose 3) (per-protocol immunogenicity cohort). 95% CI, 95% confidence interval; PD, protein D; PE, protein E; PilA, Pilin A; UspA2, ubiquitous surface protein A2. 0–2–6 group was given vaccine at 0–2–6 months and placebo at 12 months; 0–2–12 group was given vaccine at 0–2–12 months and placebo at 6 months. Number of participants with available results at each time point: between 77 and 82 in 0–2–6 group, between 79 and 87 in 0–2–12 group

Figure 6 presents the GMRs of antibody concentration at each time point after the third vaccine dose versus concentration at baseline or immediately before the third dose. These confirm the immunogenicity of the third NTHi-Mcat vaccine dose and the persistence of immune responses against each antigen. At the end of the study (18 and 12 months post-dose 3 with 0–2–6 and 0–2–12 schedule, respectively), GMRs for NTHi antigens PD, PE and PilA were at least 7.7 and the GMR for Mcat UspA2 was 1.3 in both groups (Fig. 6).

**Fig. 7:**
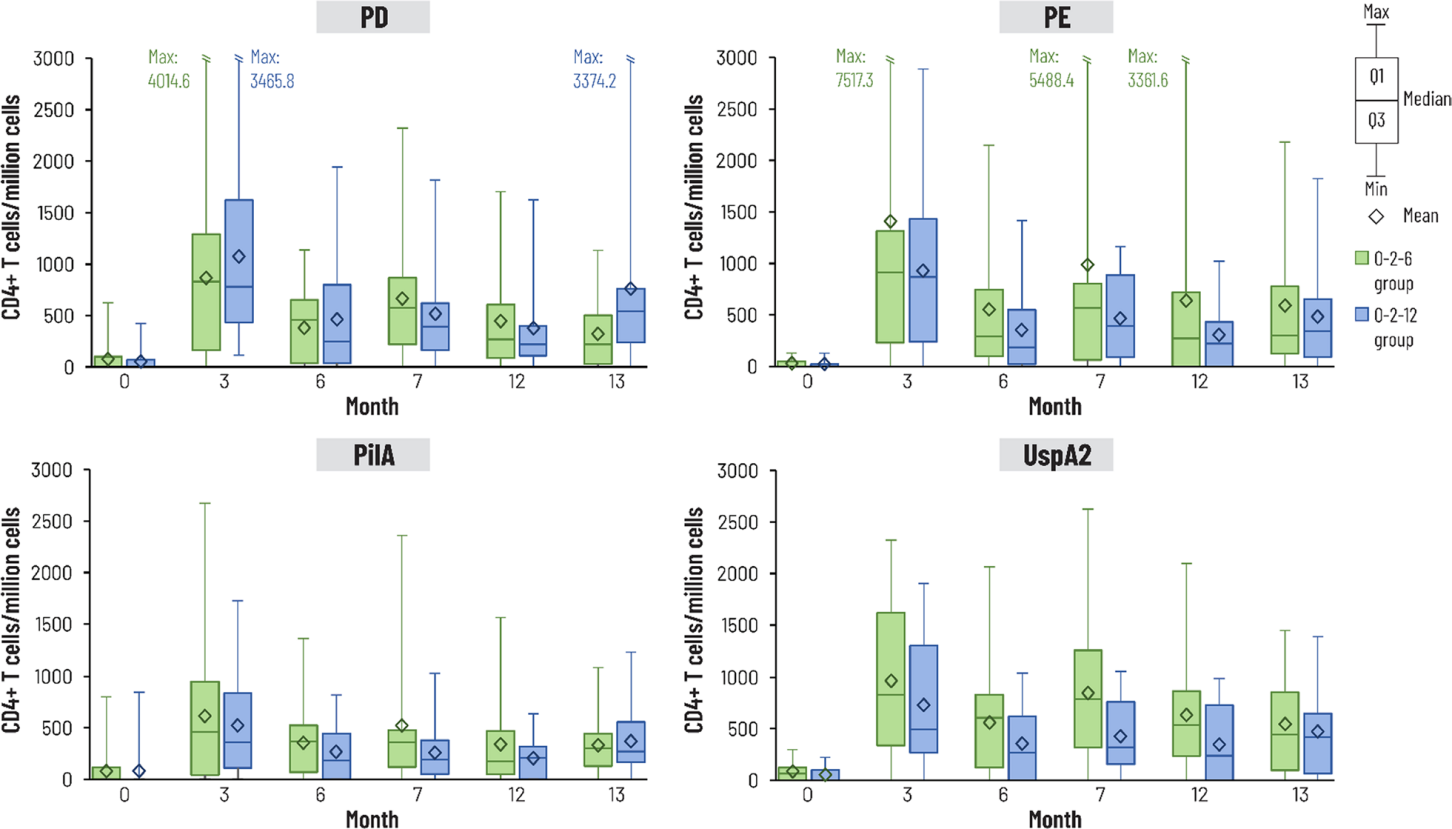
Frequency of non-typeable *Haemophilus influenzae* antigen (PD, PE, PilA) and *Moraxella catarrhalis* antigen (UspA2) specific CD4^+^ T cells expressing at least two markers among CD40 ligand (CD40L), interleukin (IL)-2, tumour necrosis factor (TNF)-α, interferon (IFN)-ɣ, IL-13 and IL-17 (cell-mediated immune response analysis subset). Median, first/third quartile (Q1/Q3) and maximum/minimum (Max/Min) percentages shown. PD, protein D; PE, protein E; PilA, Pilin A; UspA2, ubiquitous surface protein A2. 0–2–6 schedule, group given vaccine at 0–2–6 months and placebo at 12 months; 0–2–12 schedule, group given vaccine at 0–2–12 months and placebo at 6 months. Number of participants with available results at each time point: between 17 and 21 in 0–2–6 group, 17 or 19 in 0–2–12 group

Evaluation of the frequency of antigen-specific CD4^+^ T cells expressing at least two markers among CD40L, IL-2, TNF-α, IFN-ɣ, IL-13 and IL-17 did not show any specific trend during the study following each vaccine dose with either schedule (Fig. 7). CD4^+^ T cell responses were variable but higher than baseline at all time points for each antigen with both schedules. Pronounced IL-13 expression was not detected (Additional file 1: Fig. S2).

## Discussion

This is the first study in which a three-dose schedule of the investigational NTHi-Mcat vaccine was assessed in adults. No safety concerns were identified when a third vaccine dose was given to older adults with a smoking history. This is consistent with the safety of the two-dose NTHi-Mcat vaccine schedule administered to smokers or ex-smokers of similar age [15] or patients with COPD [21]. This is also in line with the safety profile of the vaccine containing only the NTHi component in older smokers/ex-smokers [16] and in patients with COPD [25].

Incidences of solicited local AEs were similar following each vaccine dose and also similar between groups. The frequency of solicited general AEs after the third vaccine dose was higher in the 0–2–12 group (53.8%) than in the 0–2–6 group (39.3%) and was also higher in the 0–2–12 group after the second vaccine dose (47.4% versus 41.3% in 0–2–6 group). The percentage of participants reporting grade 3 solicited AEs was higher in the 0–2–6 group than in the 0–2–12 group after the second vaccine dose (22.8% versus 5.2%, respectively) and overall (32% and 16%, respectively). However, the sample size was small, and the study was not powered to make formal comparisons between the two groups. Most solicited AEs were mild or moderate and grade 3 events were transient, resolving within 6 days. After the single placebo injection, frequencies of individual solicited AEs, apart from fever, were generally lower than after each vaccine dose, which may be related to more intense activation of the innate immune response by AS-adjuvanted vaccines [16, 26].

A similar percentage of participants reported unsolicited AEs within 30 days of vaccination with each schedule. After the third vaccine dose, unsolicited AEs were reported in 14.4% of the 0–2–6 group and 9.7% of the 0–2–12 group, which was in line with percentages following the placebo injection (8.2% in 0–2–6 group and 11.3% in 0–2–12 group). Unsolicited AEs causally related to vaccination were non-serious and were reported in 7% of participants in each group. Five of the related events were grade 3, two in the 0–2–6 group and three in the 0–2–12 group. In the entire study period of 24 months, 18 SAEs were reported in each group. There were three deaths: one with unknown cause in the 0–2–6 group and two (myocardial infarction and lung cancer) in the 0–2–12 group. Six participants reported pIMDs (three in 0–2–6 group and three in 0–2–12 group). None of the SAEs, deaths or pIMDs were assessed as causally related to vaccination.

Humoral immune responses against the NTHi antigens following the first and second vaccine doses in the present study displayed patterns similar to those observed previously with two doses of NTHi-Mcat vaccine or NTHi vaccine [15, 16, 21, 25]. After the third NTHi-Mcat vaccine dose, there was another peak in antibody GMC for each NTHi antigen and specific immune responses persisted with both schedules.

Specific immune responses were also observed against the Mcat antigen and, although not as strong as against the NTHi antigens, anti-UspA2 antibody concentrations remained above baseline in both groups for the duration of the study. The difference in strength of immune response may be due to the relatively high level of anti-UspA2 antibodies before vaccination, as also reported in another NTHi-Mcat vaccine study [15]. The CMI results must be interpreted with caution due to the small number of participants included in this investigation. Antigen-specific CMI responses showed high variability, as previously reported [15], with no specific trend following each vaccine dose with both schedules, although CD4^+^ T cell responses were higher than baseline at all time points for each antigen.

As already highlighted, this study is limited by its small sample size. Also, the primary safety endpoint was descriptive only and there was no alpha adjustment for multiple testing of the secondary endpoints. However, this was regarded as acceptable since this was the first study to assess the safety of a three-dose schedule of the investigational vaccine.

## Conclusions

This phase 2 study evaluated the safety and immunogenicity of a three-dose schedule of the investigational NTHi-Mcat vaccine in older adults with a smoking history. No safety concerns were identified when a third dose was administered 6 or 12 months after the first. Each NTHi-Mcat vaccine dose was immunogenic and persistent immune responses were observed after the third dose for both vaccine schedules.

## Supplementary Information

**Additional file 1: Supplementary Methods.** Inclusion criteria for enrolment. Exclusion criteria for enrolment. Gating strategy used to identify antigen-specific T cells. **Fig. S1.** Gating strategy for the ICS-9P assay using FlowJo software. **Table S1.** Percentage of participants with seropositive antibody concentrations for each vaccine antigen. **Fig. S2.** Frequency of non-typeable Haemophilus influenzae antigen (PD, PE, PilA) and Moraxella catarrhalis antigen (UspA2) specific CD4+ T cells expressing interleukin-13 (cell-mediated immune response analysis subset).

## Data Availability

Anonymised individual participant data and study documents can be requested for further research from www.clinicalstudydatarequest.com.

## Abbreviations

AE: Adverse event
AECOPD: Acute exacerbations of chronic obstructive pulmonary disease
AS01_E_: Adjuvant System containing 3-*O*-desacyl-4'-monophosphoryl lipid A
QS-21: (*Quillaja saponaria* Molina, fraction 21) and liposome (25 µg MPL and 25 µg QS-21)
CD40L: CD40 ligand
CI: Confidence interval
CMI: Cell-mediated immunity
COPD: Chronic obstructive pulmonary disease
ELISA: Enzyme-linked immunosorbent assay
EU: Enzyme-linked immunosorbent assay unit
GMC: Geometric mean concentration
GMR: Geometric mean ratio
IL: Interleukin
Mcat: *Moraxella catarrhalis*
NTHi: Non-typeable *Haemophilus influenzae*
PD: Protein D
PE: Protein E
PilA: Pilin A
pIMD: Potential immune-mediated disease
SAE: Serious adverse event
TNF: Tumour necrosis factor
UspA2: Ubiquitous surface protein A2
