# Supplementary material for: Safety and immunogenicity of three doses of non-typeable Haemophilus influenzae-Moraxella catarrhalis (NTHi-Mcat) vaccine when administered according to two different schedules: a phase 2, randomised, observer-blind study
Source: Respir Res. 2022 May 4;23:114. doi: 10.1186/s12931-022-02019-4 (PMC9069748; doi:10.1186/s12931-022-02019-4)
Supplement: Supplementary file 1 — Additional file 1: Supplementary Methods. Inclusion criteria for enrolment. Exclusion criteria for enrolment. Gating strategy used to identify antigen-specific T cells. Fig. S1. Gating strategy for the ICS-9P assay using FlowJo software. Table S1. Percentage of participants with seropositive antibody concentrations for each vaccine antigen. Fig. S2. Frequency of non-typeable Haemophilus influenzae antigen (PD, PE, PilA) and Moraxella catarrhalis antigen (UspA2) specific CD4+ T cells expressing interleukin-13 (cell-mediated immune response analysis subset). [file 12931_2022_2019_MOESM1_ESM.docx]

# Additional Information

## Additional Methods

**Inclusion criteria for enrolment**

Male or female participants aged between 40 and 80 years at the time of first vaccination were enrolled if, in the opinion of the investigator, they met the requirements of the protocol (e.g. completion of diary cards, completion of blood draws, return for follow-up visits). Participants had to have a smoking history of at least 10 pack-years. Current smokers were defined as people who were smoking or who stopped smoking less than 6 months before entry into the study and former smokers as people who had stopped smoking for at least 6 months. A pack-year was defined as 20 cigarettes smoked every day for one year and the number of pack-years was calculated as: (average number of cigarettes smoked per day times number of years smoked) divided by 20. Women of non-childbearing potential or on adequate contraception could be enrolled.

**Exclusion criteria for enrolment**

- Use of any investigational or non-registered product (drug or vaccine) other than the study vaccines within 30 days preceding the first dose of study vaccine, or planned use during the study period.
- Any medical condition that, in the investigator’s opinion, made intramuscular injection unsafe.
- Chronic administration (defined as more than 14 days in total) of immunosuppressants or other immune-modifying drugs within 6 months before the first vaccine dose. Inhaled and topical steroids were allowed.
- Administration of long-acting immune-modifying drugs at any time during the study period.
- Administration of a vaccine not foreseen by the study protocol in the period starting 30 days before the first dose and ending 30 days after the last dose of vaccine, with the exception of any influenza or pneumococcal vaccine, which may be administered at least 15 days before or after any study vaccine dose.
- Participation in another clinical study at any time during the study period, in which the participant was exposed to an investigational or a non-investigational vaccine/product (pharmaceutical product or device).
- Previous vaccination with any vaccine containing NTHi or Mcat antigens.
- Any confirmed or suspected immunosuppressive or immunodeficient condition, based on medical history and physical examination, or a history of or current autoimmune disease.
- History of any reaction or hypersensitivity likely to be exacerbated by any component of the vaccine.
- Acute disease or fever (temperature ≥37.5°C for oral or axillary route) at the time of enrolment. Participants with a minor illness (such as mild diarrhoea, mild upper respiratory infection) without fever could be enrolled at the discretion of the investigator.
- Administration of immunoglobulins or any blood products within the 3 months before the first dose of study vaccine or planned administration during the study period.
- Current alcoholism and/or drug abuse.
- Diagnosed with a respiratory disorder (e.g. asthma, COPD, sarcoidosis, tuberculosis, bronchiectasis, lung fibrosis, pulmonary embolism, pneumothorax, or physician-confirmed lung cancer).
- Significant disease (including significant neurological or psychological disorders), in the opinion of the investigator, likely to interfere with the study and/or likely to cause death within the study duration.
- Malignancies (excluding non-melanoma skin cancer) or lymphoproliferative disorders within previous 5 years.
- Pregnancy or breast-feeding. Women planning to become pregnant or discontinue contraceptive precautions.
- Any other condition that the investigator judged could interfere with study findings.

**Gating strategy used to identify antigen-specific T cells**

Fig. S1 shows GSK’s gating strategy for the ICS-9P (Intracellular Staining – 9 Parameters) assay using *FlowJo** software. The successive gating steps are as follows:

1. A time gate is created to ensure that the events are captured once the flow stream has stabilised (side scatter-area [SSC]-A versus time density plot);

2. A gate is made on singlets (doublets are excluded [forward scatter-height [FSC]-H versus FSC-A plot]);

3. A gate is made on live cells (dead cells are excluded [live-dead dye parameter versus SSC-A plot]);

4. A lymphocyte gate is created based on size and granularity (FSC-height [FSC-H] [size] versus SSC-A [granularity] plot);

5. CD3^+^ T cells are gated on (CD3 versus IFNγ plot; as the CD3 marker expression is downregulated after stimulation, a polygon gate is used);

6. CD4^+^ (CD4^+^CD8^-^) and CD8^+^ (CD8^+^CD4^-^) T cells are then gated on (CD4 versus CD8 plot);

7. Finally, analysis of activations markers (SSC-A versus CD40L [CD154], IL-2, IL-13, IL-17, TNFα, and IFNγ respectively) is made on both CD4^+^CD8^-^ T cells and CD8^+^CD4^-^ T cells.

This same strategy was applied for all clinical study samples: each sample (one subject per stimulation condition) is analysed in one file (batch) in a template using the gating strategy exemplified, from which a PDF is generated. All data files are conserved on a secured platform.

* FLOWJO is a trademark of FLOWJO, LLC

## Fig. S1. Gating strategy for the ICS-9P assay using *FlowJo* software.

**
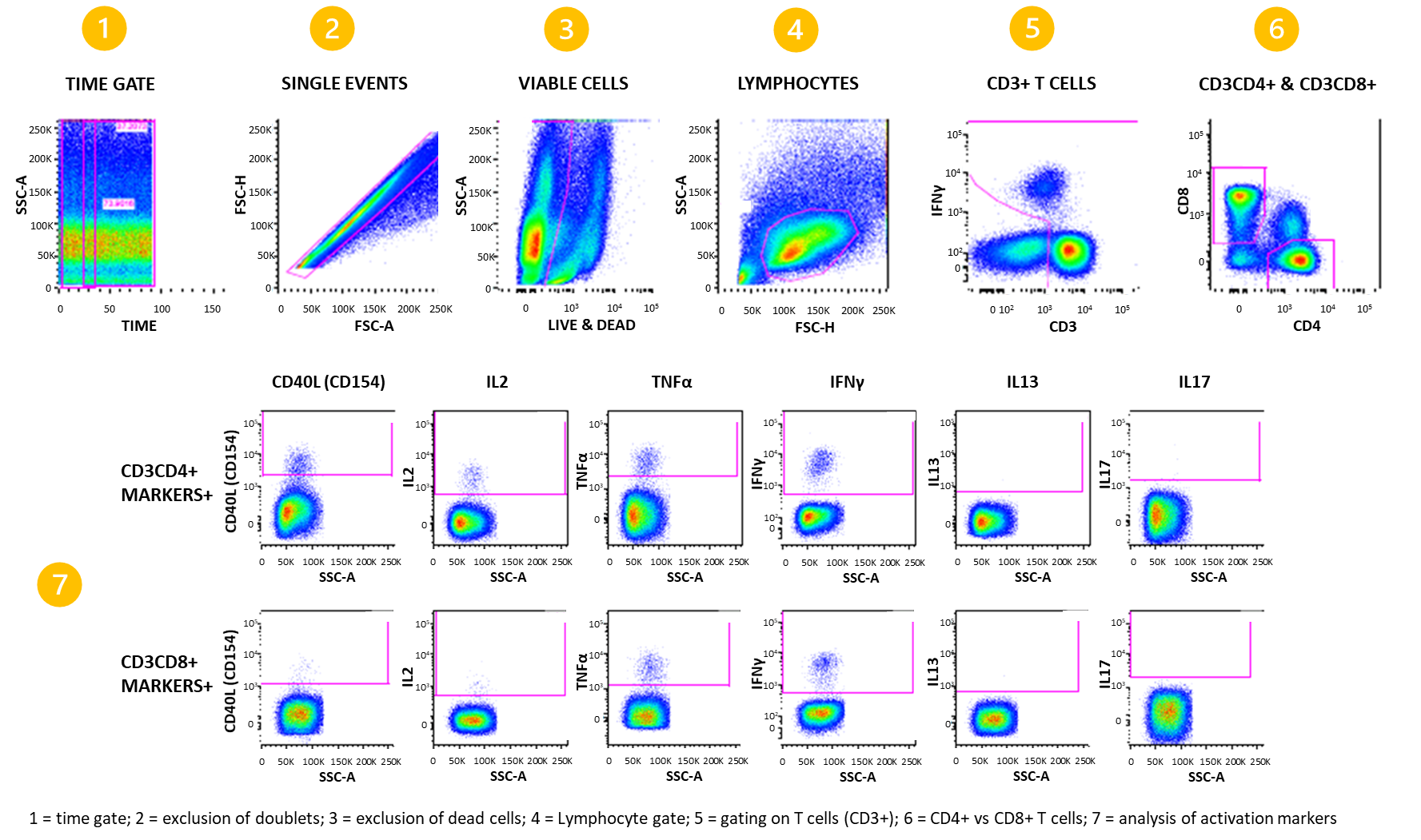
**

CD40-L, CD40 ligand; FSC-A, forward scatter-area; FSC-H, forward scatter-height; IFN, interferon; IL, interleukin; SSC-A, side scatter-area; TNF, tumor necrosis factor

## Table S1. Percentage of participants with seropositive antibody concentrations for each vaccine antigen.

| **Antigen (LLOQ)**  **Timepoint** | **0-2-6 schedule** | | **0-2-12 schedule** | |
| --- | --- | --- | --- | --- |
|  | **N** | **% (95% CI)** | **N** | **% (95% CI)** |
| PD (153 EU/mL) |  |  |  |  |
| Month 0 | 82 | 8.5 (3.5–16.8) | 87 | 13.8 (7.3–22.9) |
| Month 3 | 79 | 98.7 (93.1–100.0) | 82 | 97.6 (91.5–99.7) |
| Month 6 | 81 | 92.6 (84.6–97.2) | 87 | 92.0 (84.1–96.7) |
| Month 7 | 81 | 100 (95.5–100) | 84 | 89.3 (80.6–95.0) |
| Month 12 | 82 | 95.1 (88.0–98.7) | 87 | 81.6 (71.9–89.1) |
| Month 13 | 81 | 95.1 (87.8–98.6) | 82 | 98.8 (93.4–100.0) |
| Month 18 | 80 | 95.0 (87.7–98.6) | 84 | 94.0 (86.7–98.0) |
| Month 24 | 77 | 93.5 (85.5–97.9) | 79 | 93.7 (85.8–97.9) |
| PE (16 EU/mL) |  |  |  |  |
| Month 0 | 82 | 52.4 (41.1–63.6) | 87 | 49.4 (38.5–60.4) |
| Month 3 | 79 | 100 (95.4–100) | 82 | 100 (95.6–100) |
| Month 6 | 81 | 100 (95.5–100) | 87 | 100 (95.8–100) |
| Month 7 | 80 | 100 (95.5–100) | 84 | 100 (95.7–100) |
| Month 12 | 82 | 100 (95.6–100) | 87 | 100 (95.8–100) |
| Month 13 | 81 | 100 (95.5–100) | 82 | 100 (95.6–100) |
| Month 18 | 80 | 100 (95.5–100) | 84 | 100 (95.7–100) |
| Month 24 | 77 | 100 (95.3–100) | 79 | 100 (95.4–100) |
| PilA (8 EU/mL) |  |  |  |  |
| Month 0 | 82 | 48.8 (37.6–60.1) | 87 | 41.4 (30.9–52.4) |
| Month 3 | 79 | 100 (95.4–100) | 82 | 100 (95.6–100) |
| Month 6 | 81 | 100 (95.5–100) | 87 | 100 (95.8–100) |
| Month 7 | 81 | 100 (95.5–100) | 84 | 98.8 (93.5–100.0) |
| Month 12 | 82 | 100 (95.6–100) | 87 | 96.6 (90.3–99.3) |
| Month 13 | 81 | 100 (95.5–100) | 82 | 100 (95.6–100) |
| Month 18 | 80 | 100 (95.5–100) | 84 | 100 (95.7–100) |
| Month 24 | 77 | 97.4 (90.9–99.7) | 79 | 100 (95.4–100) |
| UspA2 (28 EU/mL) |  |  |  |  |
| Month 0 | 82 | 100 (95.6–100) | 87 | 100 (95.8–100) |
| Month 3 | 79 | 100 (95.4–100) | 82 | 100 (95.6–100) |
| Month 6 | 81 | 100 (95.5–100) | 87 | 100 (95.8–100) |
| Month 7 | 81 | 100 (95.5–100) | 84 | 100 (95.7–100) |
| Month 12 | 82 | 100 (95.6–100) | 87 | 100 (95.8–100) |
| Month 13 | 81 | 100 (95.5–100) | 82 | 100 (95.6–100) |
| Month 18 | 80 | 100 (95.5–100) | 84 | 100 (95.7–100) |
| Month 24 | 77 | 100 (95.3–100) | 79 | 100 (95.4–100) |

% (95% CI), percentage of participants (95% confidence interval); EU, ELISA units; LLOQ, lower limit of quantification; N, number of participants with available results at each time point; PD, protein D; PE, protein E; PilA, Pilin A; UspA2, ubiquitous surface protein A2. 0-2-6 group was given vaccine at 0-2-6 months and placebo at 12 months; 0-2-12 group was given vaccine at 0-2-12 months and placebo at 6 months.

## Fig. S2. Frequency of non-typeable *Haemophilus influenzae* antigen (PD, PE, PilA) and *Moraxella catarrhalis* antigen (UspA2) specific CD4^+^ T cells expressing interleukin-13 (cell-mediated immune response analysis subset).


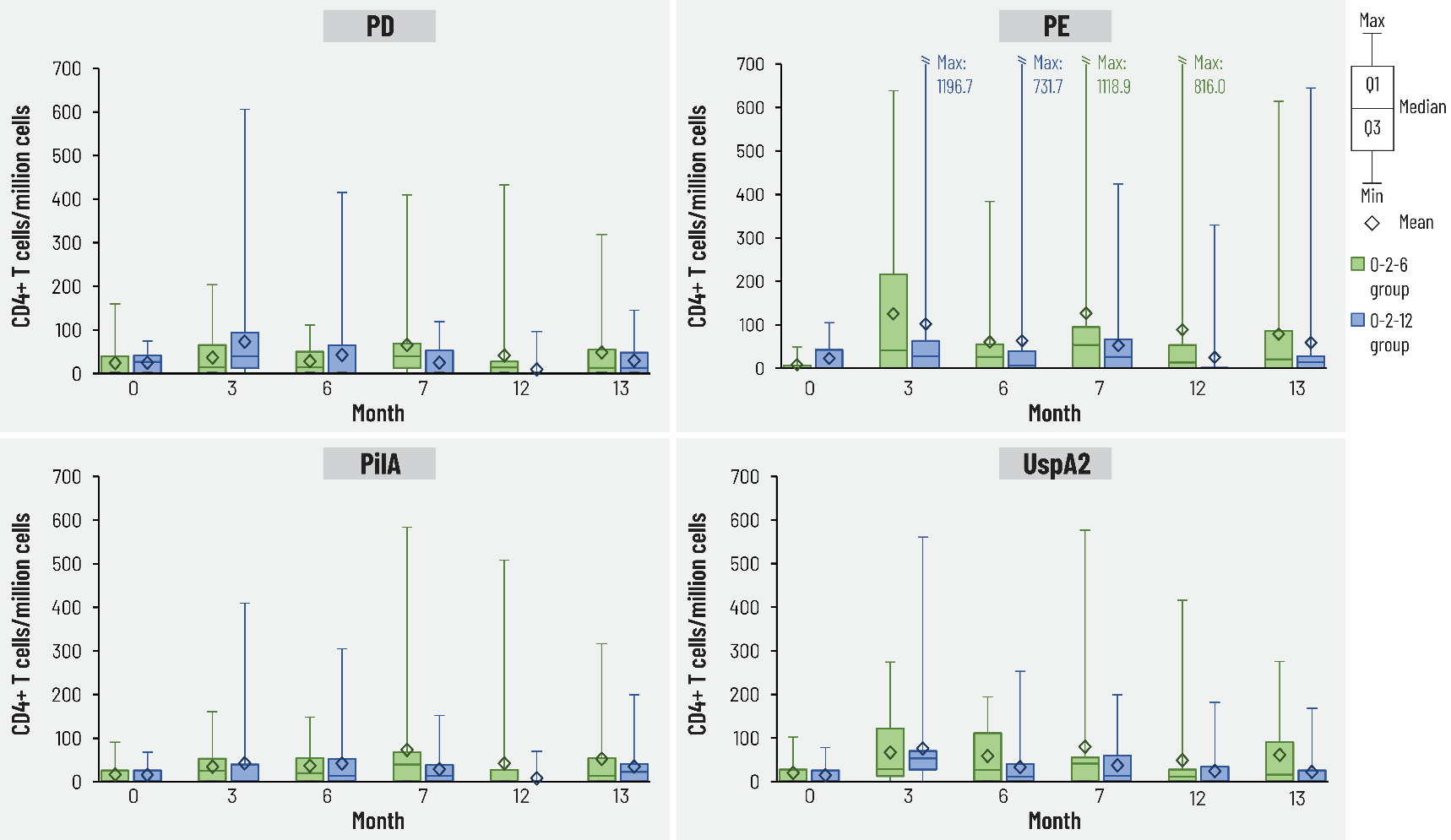


## Median, first/third quartile (Q1/Q3) and maximum/minimum (Max/Min) percentages shown

PD, protein D; PE, protein E; PilA, Pilin A; UspA2, ubiquitous surface protein A2. 0-2-6 schedule, group given vaccine at 0-2-6 months and placebo at 12 months; 0-2-12 schedule, group given vaccine at 0-2-12 months and placebo at 6 months. Number of participants with available results at each time point: between 17 and 21 in 0-2-6 group, 17 or 19 in 0-2-12 group.
